# Promoting creativity in early childhood education

**DOI:** 10.1371/journal.pone.0294915

**Published:** 2023-12-06

**Authors:** Yakup Yildirim, Yeşim Yilmaz

**Affiliations:** 1 Department of Preschool Education, Faculty of Education, Akdeniz University, Antalya, Turkey; 2 Preschool Teacher, Ministry of National Education, Alanya, Turkey; Satyawati College (Eve.), University of Delhi, INDIA

## Abstract

This study aims to find out the opinions and experience of teachers and teacher candidates on promoting creativity and creative thinking in the early childhood stage within the scope of the current preschool educational program. The method of the study is the basic qualitative research design. The study group consists of 25 preschool teachers employed in the province of Alanya in the city of Antalya, and 25 preschool teacher candidates who were students in their 3^rd^ and 4^th^ year at Akdeniz University, Faculty of Education, Department of Preschool Education. Open-ended questionnaire form was used for getting the opinions of teachers and teacher candidates. The data was analyzed within the principles of content analysis. According to the results of the study, statements on the prominence of creative thinking mainly emphasized the child being able to express her/his emotions and thoughts effectively, developing the child’s problem-solving skills, forming cause- effect relationships, and being able to create a different point of view towards events and situations. As for developing creative thinking, the opinions that come to the forefront are going on trips with the children, conducting art activities, focusing on drama activities, conducting science and maths activities based on research, and motivating children to create authentic products with different materials. On the whole, teachers and teacher candidates expressed that the present preschool educational program has positive contributions to promoting creative thinking in children.

## Introduction

What kind of atmospheres and situations in class and out of class should we create or what should we do to discover and promote the real potential of children? We have tried to seek answers to these questions from those closest to the child. Creativity may emerge when the child has the opportunity to combine different experiences in appropriate situations especially in the preschool period. This may sometimes display itself while creating a solution to a simple problem or when obtaining new knowledge by using higher skills. Creating an environment which enables the child to develop a new point of view is a leading element of creativity. Preschool is a stage during which the creativity of the child is at its peak as they have unlimited imagination.

The preschool stage, which is defined as the stage from birth to the time the child starts primary education, and a time when the child acquires the psychomotor, social- emotional, cognitive and linguistic development that will play an important role in children’s life, and a developmental and educational process during which the character of the child is shaped with the education provided by the parents and pre- school institutions [1–6] is the most critical period in life as it affects the future life of the child in terms of knowledge, skills, gaining habits and developing these traits. The preschoolperiod is a stage when mental development and synaptic connections are experienced the fastest and highest [7]. Mental development plays an important role during the preschool stage for the cognitive, linguistic, motor, social and emotional development of children.

Children grow rapidly in the preschool stage—the first six years of their life display rapid results in developmental areas [8]. This enables the child to realise herself/himself and become a productive member of society. The preschool period is the stage which is most affected by environmental factors. In this respect, the environment affects the preschool child and facilitates learning motivation for children who are in this stage. The child’s ability to discover and learn is closely related to how supportive the child’s environment is, and which opportunities are presented to the child [7].

The child, who matures and becomes competent rapidly, realises her/his own potential and starts to become a productive individual. Creativity has a prominent impact on how the child develops herself/ himself. Creativity and judgement skills enable individuals to consider problems using different views, to create new products as well as enabling them to reach a decision by forming cause- effect relationships [9]. It is necessary to create new ideas and consider events in a different way, and create new solutions to a problem. It is also important to respect different ideas [10]. It is going beyond the presented knowledge in order tocreate something authentic by using methods which are not traditional. It is defined as the skill to create innovative and authentic solutions to problematic situations by realizing problems and shortcomings within the light of experiences [11], and it may be said that it makes the child self- confident and independent and enables her/ him to develop herself/ himself and the environment, makes the child responsible towards her/his environment, makes them productive and sensitive individuals. Creativity can be enhanced by creating connections between similar or different areas [12]. Preschool children may activate their creative thinking skills when they use an object for a different purpose, when they find an extraordinary solution to a problem, while displaying motor skills, when day dreaming, while forming an emotional relationship with a peer or an adult, or in other situations which require a creative process [13].

Children who have suitable conditions for using and practicing their creative thinking actively may strengthen their cognitive skills. These conditions also contribute to the children’s social skills development such as discovering their emotions and values, understanding their own cultures and other cultures, thinking, and communicating with others [14]. Thus, different teaching approaches that will increase children’s motivation and cultural understanding could support creativity [15].

Creativity is a phenomenon needed and used in all stages of life, is a prominent factor in the development and advancement of society. In societies which have individuals who have high levels of creativity and who can use creative thinking effectively, the level of welfare increases and the opportunities for people depending on their interests and talents are equally higher. There is a positive relationship between the educational backgrounds of people and the increase in their creativity. In order to maintain progress, guarantee advancement and to have a good place in life, individuals need to get the opportunities to strengthen creativity both in the family and at home starting from the preschool stage. In an educational environment which is based on rote learning and which is teacher- centred, promoting creativity and creative thinking is more difficult compared to a child-centred environment [16].

Teachers who can create a child-centred environment and processes in which the children can develop their creativity contribute to the development of the children in all aspects as well as playing a prominent role in the progress and development of the society in which the children live. Thus, along with the development of creativity and creative thinking, some inventions result in increase in production and the economic situation of the society. Similarly, life standards increase in a society which has a developing economy. Consequently, promoting creativity in a society which lacks productive skills can be difficult [17]. The technological infrastructure, knowledge and skills of integrating technology into teaching and learning practices, and students’ creative skills of using technology is essential to promote higher thinking skills (i.e. creativity) [18].

Individuals who can think creatively become individuals who are open to change as they can adapt to the rapidly changing world. There is a positive correlation between the level of development in a country and the creativity and creative skills of the people in that society. In order to promote the development of a country, the development of creativity should be facilitated by focusing on production and innovation in different areas [19]. The adaptive skills may involve having cultural understanding of inclusive education, not only integrating children into the classroom, but also having a teaching program that will support children with special educational needs in creativity [20].

To promote creativity and creative thinking important skills for both the individual and the society, families and teachers have important roles. The family also has a prominence for developing creativity and creative thinking in children along with teachers. There are differences between the educational backgrounds of families, and this may hinder creativity in some situations. Families may be asked to help children concerning this topic by offering training to parents and educating them on creativity and creative thinking [21]. It is seen that children whose creativity is supported in the family environment offer different ways of solutions while expressing their emotions and thoughts, discover new games, are curious and are interested in travelling and observation [16].

Teachers and families may offer opportunities to children to promote their creativity and creative thinking by considering the traits that preschool children display. As the way each individual shows her/ his creative potential, and the way this potential is supported may display differences. The opinions of teachers and teacher candidates on how they discover and support the creativity of children is very important. Therefore the best way to understand these thoughts is to analyze the explanatory information they would express qualitatively. The aim of this study is to determine the prominence of creativity in preschool education, to determine the creative skills of children as well as making evaluations on what kind of studies should be conducted to develop creativity, and to determine methods and suggestions on developing creative thinking. For this purpose, answers were sought to the following questions:

- Why are creativity and creative thinking important in preschool education?
- What should we do to promote the creativity and creative thinking of children in the preschool stage?
- What are your in-class and out of class activities that you use to promote the creativity and creative thinking of preschool children?
- How did the 2013 Preschool Education Programme contribute to the development of creativity and creative thinking of children?

## Materials and method

### The research design

This study, which has been conducted to determine strategies to promote creative thinking in the preschool stage, and to create suggestions for solutions, used the basic qualitative research design, which is a qualitative research pattern. Basic qualitative research aims to find out how participants comprehend their experiences within the scope of the topic studied, and which meanings they place on their experiences [22]. Thus, this method was preferred in this study in order to determine feelings, thoughts, perceptions and experiences of teachers and teacher candidates on the prominence of creativity and the promotion of creative thinking in the preschool stage, and to study their opinions in more detail. The open-ended questionnaire template which was developed to get written opinions was used for data collection. A comprehensive literature review was conducted for the study to reach its aims. In addition, the conceptual structure of the subject was stated within the framework of the aims and limitations of the study. Following that, open ended questionnaire forms were prepared for both teachers and teacher candidates as appropriate to the aims of the study. Thus, the purpose was to study in detail the awareness of the participants on the prominence of creativity in the preschool stage and developing creativity as well as the methods they used for this purpose.

### The study group

The study group consists of preschool teachers who are employed at preschools in the province of Alanya in the city of Antalya, and preschool teacher candidates who were students in their 3rd and 4th year at Akdeniz University, Faculty of Education, Department of Preschool Teaching. The 25 preschool teachers and 25 teacher candidates who met this criteria and who participated in the study group were determined by using the purposive sampling method [23]. The main purpose for preferring this sampling method is that the participants are chosen according to certain criteria determined by the researchers beforehand [24]. When choosing the participants among the teacher candidates attending their third and fourth year at university, the main determining factor was that they had taken the classes which were ‘creativity, school experience and/ or teaching practice’. Another point which was given priority during the study was ensuring that preshool teachers and teacher candidates gave sincere answers to the questions which were included in the data collection tool, and which were directed towards the experiences and practices of the participants. For this reason, special care was taken to make sure that the preschool teachers participating in the study had spent a certain amount of time working with the children so that they were able to get to know the children better, and that they could express their experiences more clearly. In addition, special care was taken to ensure that the professional seniority of the teachers were different from each other and that met the desired criteria in terms of seniority (See. Table 1). The data on the professional seniority of the preschool teachers participating in the study are presented in the table below:

**Table 1:** The professional seniority of the teacher participants.

Professional Experience	Teacher 1	Teacher 2	Teacher 3	Teacher 4	Teacher 5	Teacher 6	Teacher 7	Teacher 8	Teacher 9	Teacher 10	Teacher 11	Teacher 12	Teacher 13	Teacher 14	Teacher 15	Teacher 16	Teacher 17	Teacher 18	Teacher 19	Teacher 20	Teacher 21	Teacher 22	Teacher 23	Teacher 24	Teacher 25
**1–5 years**								**X**	**X**										**X**		**X**				**X**
**6–10 years**		**X**											**X**		**X**		**X**	**X**				**X**			
**11–15 years**			**X**	**X**	**X**	**X**				**X**	**X**			**X**		**X**									
**16 years and up**	**X**						**X**					**X**								**X**			**X**	**X**	

### Data collection tools

When the data collection tool of the research was being prepared, the related regulations and the Ministry of Education Preschool Educational Program [7] was studied as well as the related literature review. As a result of the theoretical knowledge in the related literature and the interviews conducted with experts, ‘open-ended questions were prepared’ in order to determine the opinions of teachers and teacher candidates for the aims of the study. The steps to develop the data collection tool is listed in Table 2. Due to the pandemic, the opinions of teachers and teacher candidates were obtained using online methods. After the subject and aims of the study were explained to teachers and teacher candidates, open-ended online questionaire forms were sent to volunteers, and they were asked to answer the questions in the data collection tool. The participants were told that it was prominent that they put emphasis on their personal experiences and pay attention to their practices or future practices while offering suggestions. The first part of the data collection tool includes the personal information of teachers and teacher candidates. The second part of the data collection tool focuses on the prominence of creativity and creative thinking in the preschool stage. The third part contains what should be done in order to promote creativity and creative thinking during the preschool stage while the fourth part focuses on in- class and out of class activities that affect creativity and creative thinking. The fifth part includes the suggestions of preschool teachers and teacher candidates on the contribution of Preschool Educational Program on the development of creativity and creative thinking in children.

**Table 2:** The steps used to develop the data collection tool.

***Step 1***: The open-ended questionnaire was structured with four questions
***Step 2***: The open- ended questionnaire form was examined by 3 experts who were experienced in qualitative research. The statements for the items were changed based on the views of the experts. The new forms were re-sent to the experts for a second examination.
***Step 3***: The questions in the open-ended questionnaire form were directed to 3 teachers for the pilot study. Some changes were made in statements which were unclear.
***Step 4***: Following the pilot study, the questions in the open-ended questionnaire were examined by 3 experts who were experienced in qualitative research for the correction of statements which were unclear.
***Step 5***: The final form of the open-ended questionnaire form was prepared in order to be used in the study.

### The data collection stage and ethical procedure

During the data collection process, it was stated that teachers and teacher candidates were to pay attention to certain criteria while filling in the open-ended questionnaire forms.

1. The open-ended questionnaires were sent to teachers via online methods as it was impossible to conduct face-to-face interviews with the participants because of the pandemic. These open-ended questionnaire were conducted between March 15^th^ 2021 and June 28^th^ 2021.
2. Before filling in the open-ended questionnaire forms, written consent form was signed by adult participants to make sure that they are aware of the ethical issues.
3. Each teacher and candidate teacher was told that that codings would be used instead of their names, and that their real names would not be used so as to ensure that the participants would answer the research questions sincerely.
4. The data obtained in the pilot study was not included in the final findings of the study.
5. Codings were given to each participant, and their names and schools were kept secret so that teachers would give sincere answers to research questions. The abbreviations used for the coding for the reporting were made as:
  - Teacher 1 (T1)   - Teacher Candidate 1 (TC1)
  - Teacher 2 (T2)   - Teacher Candidate 2 (TC2)

This study is approved by Social Sciences and Humanities Scientific Research and Publication Ethics Committee of Akdeniz University.

### Data analysis

**Fig 1:**
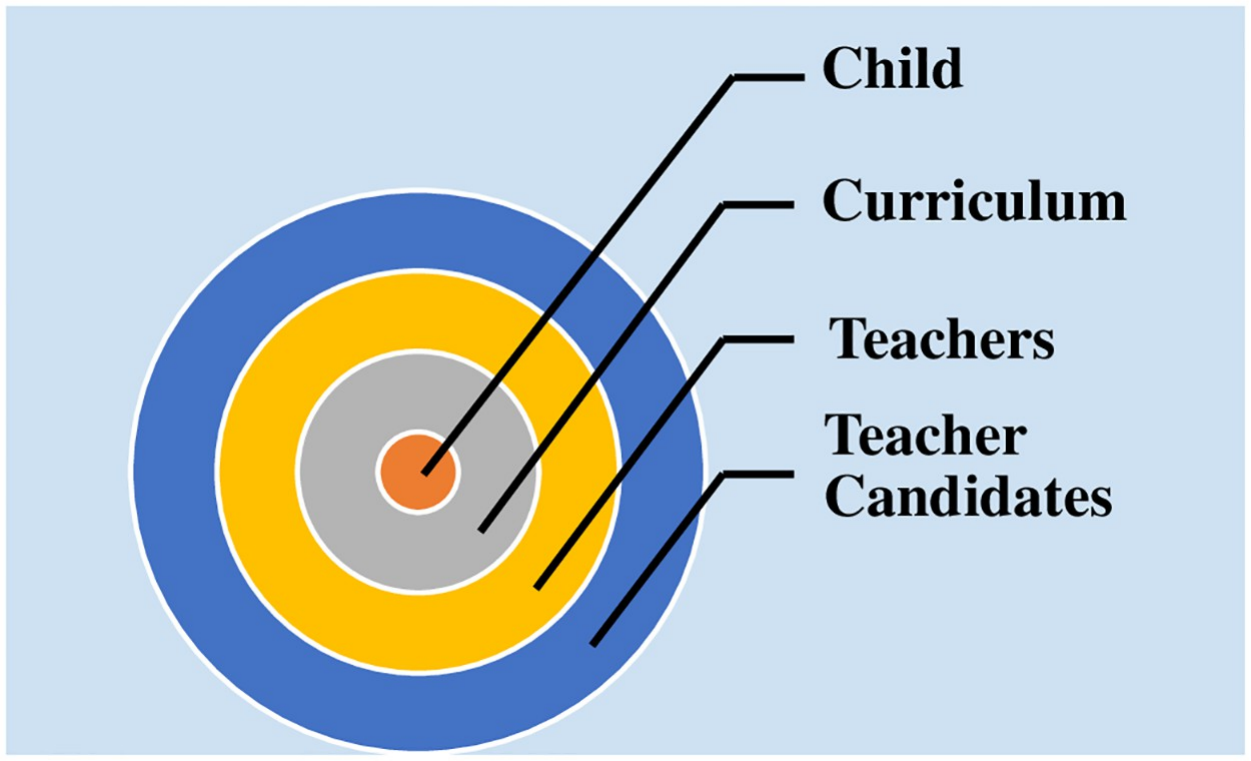
Child-centred data analysis.

The content analysis method was used for analyzing the study data of the participants in the open-ended questionnaire form by applying a child-centred data analysis method (see Fig 1). The main purpose of content analysis is to reach concepts and connections that would assist in explaining the comprehensive data obtained in the study. Data, which is summarized descriptively and commented on broadly, is studied in detail using content analysis, and new concepts and connections are discovered. The basic process here is to gather related data within the framework of specific themes and concept and present the data in a meaningful and organized way [24, 25]. The themes were created according to the results of the analysis obtained using content analysis. The codes that emerged during creating the themes were presented to the opinion of an expert for reliability (Reliability = consensus / consensus + disagreement) as suggested by Miles and Huberman [26]. The reliability of the experts and researchers for the relationship between the codes and the themes was calculated as 89%. The themes which were created were presented as items in findings, and the information on the preschool educational program and regulations were added to the end of each theme in order to compare the data obtained from participants for each theme. Statements were presented in the findings of the study in order to maintain the reliability of the study.

### Findings

This part presents the findings obtained from the participants within the framework of the themes of the study. Themes and subthemes were analysed by presenting the tables for the subthemes of each theme. The findings of both preschool teachers and teacher candidates were presented after the tables.

### Theme 1. Awareness on creativity

- **The Opinions of Preschool Teachers on Theme 1**

When Table 3 is studied, it is observed that preschool teachers participating in the study mentioned the following categories on the prominence of creativity and creative thinking in the preschool stage:

a) The relationship between imagination and creativity enables the child to express her/ his thoughts
b) It enables the child to gain communicative skills
c) It develops the child’s problem-solving skills
d) It enables the child to create cause- effect relationships
e) It enhances the child’s curiosity and the wish to discover
f) Contributes to scientific research
g) Enables the child to develop a different point of view
h) It provides hands-on learning to the child

It may be said that the categories least mentioned by the teachers are that it makes each child feel special, it enables self-realization, and it contributes to the social skills development of the child.

**Table 3:** The teachers’ thoughts on creativity and the prominence of creative thinking in the preschool stage.

Theme 1: Awareness on Creativity
**Subthemes for Theme 1**
Supporting imagination
The skill to express emotions and thoughts
Problem solving
Vocabulary
Feeling special
Self-realization
Curiosity and Discovery
Looking at events with a different view
Effective Learning
Support for Social Skills

### A general evaluation on subthemes of the first theme

When the opinions of preschool teachers on the theme ‘the prominence of creativity and creative thinking’, are studied it is observed that teachers believe that creativity and creative thinking develop the most when children use their imagination and the power of thought, and that the preschool stage was a very important stage for developing these skills as their imagination is at its peak during the preschool stage. When the teachers were stating their opinions on the prominence of creativity and creative thinking during the preschool stage, they focused on the fact that it would help children to express their emotions and thoughts, and help them in gaining communicative skills. They have also stated that the problem-solving skills of children would develop, and that they could understand cause- effect relationships between events in this way. Examples for the teachers’ opinions on the first theme and its subthemes are presented as follows:

- Creative thinking and using imagination. This stage, in which imagination is unlimited, is a stage that should not be missed to promote creativity and creative thinking skills. For most children, creativity is at its peak before the age of six (T1).
- The preschool stage is a world during which imagination and cognitive skills are unlimited. Developing this world starts with discovering the creative thinking of the child (T14).
- It is important because the children can learn to express themselves (T19).
- Children who have creative thinking skills also develop their communication skills (T23).
- It is important to promote creative thinking so that they can find authentic solutions to problems (T8).
- Helping students to form cause- effect relationships plays a very important role in children’s discovering their talents. Children who have creative thinking skills also develop their skills for communication, problem solving, practice, following instructions, and starting and maintaining projects (T23).

- **The Opinions of Preschool Teacher Candidates on the First Theme**

The opinions of teacher candidates’ preschool children on the prominence of creativity and creative thinking in preschool children are presented below:

When Table 4 is studied, it may be said that the preschool teacher candidates participating in the study mostly focused on the following categories on the prominence of creativity and creative thinking in preschool education:

- It helps the child to create a different point of view
- It develops the problem-solving skills of the child
- It affects the child’s life
- It develops the child’s imagination
- Creativity and creative thinking are very important in the preschool stage

**Table 4:** The opinions of teacher candidates on the prominence of creativity and creative thinking in the preschool stage.

Theme 1: Awareness for creativity
**Subthemes for the First Theme**
Different point of view
Discovering oneself
Problem solving
Promoting imagination
Effect on the development of the child
Support for the child’s learning
Understanding the child
Being able to express oneself

The categories least mentioned by teacher candidates for the theme ‘the prominence of creativity and creative thinking’ were the following:

- It enables the child to discover and get to know herself/ himself
- It contributes to the developmental aspects of the child
- Creativity contributes to the child’s learning
- It helps us to understand the child
- It enables the child to express her/ his feelings and thoughts

### A general evaluation of the subthemes of the first theme

In their opinions on the theme ‘awareness for creativity’ teacher candidates drew attention to the fact that creativity and creative thinking was an important factor in helping the child realize her/ his potential, and in strengthening the child’s self-realization. Teacher candidates, who focused on the fact that creativity and creative thinking developed the imagination and the potential of the child, also mentioned the contribution of creativity and creative thinking on the social and cultural life of the child. The fact that creative thinking makes life easier for the child and would provide proactive conditions to the child in social life and in problematic situations in the future is the opinion of teacher candidates that stands out in the subthemes of the first theme. Example statements of teacher candidates that express that developing creativity and creative thinking presents positive contributions to different developmental aspects of the child are as follows:

- If we can help them to discover their creativity and develop this potential in this stage, they may create more practical solutions to situations they may experience in the future and have different content (TC4).
- The schemes created by the child in this stage contributes to the child’s creativity in the future (TC1).
- The child discovers and gets to know herself/ himself with creative thinking (TC2).
- The child may discover herself / himself by thinking differently (TC9).
- It is important as they can find different and authentic solutions to problems they may encounter throughout their lives (TC17).
- The activities which are used during this stage affect the creative skills of the child in the coming years (TC12).
- It is effective for the cognitive, social, emotional, and psychomotor development of the child (TC11).

### Theme 2. Promoting creativity

- **The Opinions of Preschool Teachers on Second Theme**

Table 5 presents the suggestions and the subthemes created by preschool teachers for the theme ‘promoting creativity’. Preschool teachers suggested creating environments in which the child can ask questions and express herself/ himself, providing them with creative environments, having structured activities, motivating children for creative thinking by asking them open-ended questions, creating environments that would arouse interest, designing activities and games, and enabling children to discover themselves and their environment for promoting creativity. When the sub themes for the second theme are studied, the topics least mentioned by teachers were that they need to discover the inner world of the child, conducting attention and coding activities, and giving children some responsibilities in the family.

**Table 5:** The teachers’ opinions on supporting the creativity and creative thoughts of children.

Theme 2: Promoting Creativity
**Subthemes**
Creating suitable environments
Creative environments and structured activities
Theatre and drama
Open-ended questions
Different environments and activities
Creating awareness
Discovering your own potential
Getting into the world of the child
Solving problems together
Giving responsibilities to children
Promoting vocabulary
Stereotyped attitude
Respect to individual differences
Gamification
Promoting attention

### A general evaluation of the subthemes of the second theme

The suggestion most emphasized by teachers for promoting creativity in the preschool stage is the need for creating an environment that keeps the curiosity of the child active and enables the child to express herself/ himself. It was stated that a process in which children are asked open-ended questions that would make them think would contribute to promoting creative thinking in children. Teachers stated that activities which are not structured and ones which the child could shape using her/ his interest are more functional, and that they are an important factor that supports creativity. It may be said that especially manipulative materials enable children to think in different ways. The prominence of games, the fact that games open the doors to the inner world of children, that children may face different challenges and create authentic solutions through games are among the suggestions of teachers. The following statements of teachers draw attention in their suggestions for promoting creativity and creative thinking in children:

- Creativity develops in environments in which the child can express herself/ himself with self- confidence and show her/ his curiosity (T1).
- Children should be supported to express themselves by asking open-ended questions (T15).
- Open-ended questions, art, music, movement, and dance activities enhance creative expression. They should be given opportunities to create their own stories by looking at illustrations in books. Children may create new objects using their imagination by using games such as puzzles and building blocks. Using play dough may be effective in gaining creative skills by creating the objects in their imagination (T1).
- We may give them different materials and ask them to create new things, or we may give the same materials at different times and expect them to create different things each time (T3).
- We may encourage them to think by asking open-ended questions (T22).
- In order for them to discover creative thinking, games and activities should be designed to increase their curiosity (T2).
- Children should be provided environments that can arouse their curiosity. We should trigger their curiosity by offering opportunities for play and give them a chance to experience their creativity (T10).
- We should open a door to their inner world by using games and determine their needs (T5).
- We should not stereotype them while they are making these discoveries (T16).

- **The Opinions of Preschool Teachers Candidate on the Second Theme**

The opinions of preschool teacher candidates on promoting creativity and creative thinking in preschool stage children are presented below:

When Table 6, which presents the suggestions of teacher candidates for promoting creativity and creative thinking in children, is studied, the suggestion that is most emphasized is the need to offer an environment of freedom to the children. It is emphasized that creating a rich environment by presenting different materials to children is another important factor that promotes children’s creativity. Another major opinion of teacher candidates for the second theme is creating authentic activities for children and providing hands-on learning.

**Table 6:** The opinions of teacher candidates on promoting creativity in children.

Theme 2: Promoting creativity
**Sub Themes**
Freedom
Fruitful environments and materials
Problem solving
Being able to express oneself
Motivating students to think
Authentic and individualized activities
Hands-on learning
Caring for different thoughts
Promoting imagination

### A general evaluation of subthemes for the second theme

Creating a suitable environment in which the child can think freely was greatly emphasized by teacher candidates as a suggestion for promoting creativity and creative thinking in children. Having different materials that motivate children to think in a different way may be stated as another suggestion that supports creative thinking. Example suggestions by teacher candidates for adding variety to materials, the quality of the questions to be asked, the children participating actively in the learning process, guiding children to create solutions to problematic situations are as follows:

- We may design and implement activities in which the children can use their imagination (TC4).
- We must give them opportunities to discover without intervention. We should help them with hands-on learning (TC11).
- We should motivate them to use hands-on learning (TC11).
- We may ask children divergent questions and motivate them to think and develop their creativity (TC7).
- Asking them questions directed at their creativity while conducting activities in class (TC19).
- We must present different stimulus to motivate the child (TC1).
- It may be necessary to conduct different activities with children using different materials. Learning centres at nursery schools are in direct proportion with this topic (TC8).
- We must respect children’s thoughts and ideas and pay attention to what they wish to do (TC13).

### Theme 3. Strategies for promoting creativity

- **The Thoughts of Preschool Teachers on the Third Theme**

Table 7 presents the strategies of preschool teachers for promoting creativity and creative thinking. It is observed that for the third theme the teachers mainly drew attention to the following categories:

a) Enabling the children to express themselves by asking open-ended questions
b) Making use of art activities, and using activities different from standard ones
c) Enabling the children to create authentic products by using different materials
d) Enabling the students to express their emotions and thoughts individually during Turkish language classes
e) Using structured and semi-structered activities
f) Using different methods and techniques in activities
g) Enabling children to express themselves through drama and game activities
h) Using science and math activities
i) Making use of out- of- class activities
j) Observing children during play and while they are not playing
k) Motivating children to carry out activities with their families in the home

**Table 7:** The strategies of preschool teachers for promoting creativity and creative thinking in the preschool stage.

Theme 3: Strategies for Developing Creativity
**Subthemes**
Open-ended questions
Breaking the mold
Creating authentic products
Using different methods and techniques
Drama and games
Science and maths activities
Outdoor activities
Child yoga
Motivating children to think
Being a good observer
Guiding students
Music and rhythm activities
Cooperation with families

### A general evaluation of the subthemes for the third theme

Teachers have emphasized that acting according to standard practices for in-class and out-of- class activities for promoting creativity hinders creative thinking, and that it is necessary to conduct activities with which the children can reflect their individual performance to the maximum, either during in-class or out-of- class activities. Teachers mentioned the prominence of trips and observation in out-of- class activities and stated that it would be useful to talk to the children about the activities following practice. They stated that using techniques such as scamper, brainstorming, dramatization that attract the attention of children and enable them to think in a different way in in-class activities should be used. Examples for the teachers’ statements for the third theme and its subthemes are as follows:

- Asking children for their opinions, asking open-ended questions, creating a model, praising creative thinking. Organising out-of- school trips and observations, and later chatting to the students about what they have seen and learnt (T8).
- I would encourage them to express themselves by asking open-ended questions during in-class activities and out-of- school activities (T2).
- I would make them create products using their creativity by using natural materials such as fabric, pinecones and twigs during art activities (T15).
- During classes I use techniques such as games, drama, scamper, and brainstorming (T8).
- I introduced them to activities that would motivate them to do research and create what they think. (STEM activities, coding, algorithm, recycling, ecology and nature activities, the Young Inventor and his Inventions, drama and the Orff approach, audio stories, games, scamper activities etc.) (T16).
- We frequently make use of experiments and maths activities (T1).
- Patterns with buttons of different sizes, measuring the length of objects, finding pairs, ordering, making comparisons. Science and nature studies in the garden, creating appropriate environments for them to study and discover stones and leaves (T10).
- They should be allowed to act freely and flexibly in the classroom without being dependent on a model, with the guidance of the teacher (T7).
- Preparing comprehensive activity plans that enhance creativity instead of steoretype activities (T17).
- Families should accept that each child in the family is an individual, determine targets parallel to the interests and talents of their children. In addition, they may cooperate with teachers to conduct activities that reinforce the school program and that are related to real life. These activities should be conducted starting from simple to difficult ones, and from the known to the unknown (T23).

- **The Opinions of Preschool Teacher Candidates on the Third Theme**

The strategies of preschool teacher candidates on developing creativity and creative thinking in preschool children are presented below:

When Table 8 is studied, it is observed that the strategies most suggested by preschool teacher candidates for promoting creativity and creative thinking in the preschool stage are taking children on trips, conducting art activities, carrying out drama activities, making Turkish language activities, conducting maths and science activities, and focusing on activities children have at home with their families. The least mentioned suggestions are not interfering when children are conducting activities, carrying out comprehensive activities with divergent questions, and motivating students to different areas of interest.

**Table 8:** The strategies of preschool teacher candidates on developing creativity and creative thinking in preschool children.

Theme 3: Strategies for Developing Creativity
**Subthemes**
Organising trips
Art facilities
Drama activities
Turkish language activities
Maths and science activities
Hands-on learning
Activities to promote discovery
Offering an environment of freedom
Divergent questions
Using objects in different ways
Supporting areas of interest
Motivating students to ask questions
Parent- school cooperation

### Evaluation of the subthemes of the third theme

Giving prominence to activities children conduct with their families attracts a lot of attention among the strategies teacher candidates have suggested for developing creativity. Another major suggestion of teacher candidates is that supporting children with different activities may enable them to think in different ways. Teacher candidates have suggested that motivating children to ask questions, and using techniques that promote creativity such as completing stories may enable children to ask different questions and enhance their creativity. Examples for the statements of teacher candidates are presented below:

- Games to develop the creative sides of children may be designed by using kitchen tools in the home, or parents may make cookies of different shapes with the children (TC5).
- Activities that are mostly based on the choices of children should be conducted. Families should read story books at home with the children, and later ask child to narrate the rest of the story, or ask them to change the ending of the story. Parents may make drawings with the children or may build towers with toys (TC13).
- Drama activities enable children to use creative thinking. These activities develop their way of thinking by causing children to use improvisation (TC3).
- Drama activities may be conducted by planning improvised activities on a certain topic (TC7).

### Theme 4. Creativity in the program

- **The Opinions of Preschool Teachers on the Fourth Theme**

When teachers were asked their opinion on the elements in the preschool educational program that supported the creativity of children, they stated that on the whole, the program enabled children to reflect their individual traits. They have also reported that the flexibility of the program enables them to restructure the program according to the individual differences of children, and that this offers them a chance to support their creativity. It may be said that teachers consider the preschool educational program as one that supports the children’s feeling of discovery and self-awareness. The teachers’ opinions on the fourth theme are presented below:

- It is child-centred. Children experience meaningful hands-on learning instead of rote learning. In this way, creativity is always active. The flexible program enables necessary changes in the educational process depending on daily and momentary changes that may arise. As individualism is the most prominent element, the program is created by taking individual differences into consideration as appropriate to the needs of the children. In this way, the differences, creativity, interests and needs of each child make each children unique (T2).
- The program basically has a structure that supports creativity and aims to strengthen it. However, the shortcomings in practice (physical shortcomings, the attitudes of teachers, the attitudes of school administration and families etc.) makes it difficult to reach goals or hinders it (T6).
- The program enabled the child to participate actively in the learning process, and encouraged the child to learn by asking questions, doing research, making discoveries, and playing games. It offered the children the necessary opportunities to express themselves authentically, and in different ways in environments which are appropriate for the learning needs and learning styles of each child (T16).
- The effect of the 2013 Preschool Program on the development of children’s creativity and creative thinking is great. As it is a flexible program, it enables teachers to plan according to the interests and talents of children, the cultural traits of the environment and the self-awareness of the children (T23).
- It develops the imagination, creative and critical thinking skills of children as well as their communication skills and their potential to express their feelings (T25).

- **The Opinions of Preschool Teacher Candidates on the Fourth Theme**

When preschool teachers candidate were talking on the advantages of the preschool educational program that supported the creativity of children, they focused on the fact that the program supported the development of children in all aspects. They stated that as the program is student- centred, it is a prominent factor in supporting the children’s creativity. The opinions of teacher candidates on the fourth theme are as follows:

- The 2023 preschool program is a program that considers children with all of their aspects and supports children’s development in all ways. Since this program is student-centred, it gives children the chance to express themselves, and to state their opinions freely. Consequently, this situation contributes positively to children’s creativity (TC5).
- In this program, activities are prepared as student- centred activities as appropriate to the program, and then put into practice. Chatting to the children about the activities prior to practice and asking open-ended questions to children following activities may give us clues on how their creativity is developing (TC7).
- This program contributes to the progress of children’s creativity by enabling the children to receive better education as it leads teachers and candidate to the right path (TC1).
- The 2013 preschool program was prepared by studying different programs that would contribute to different types of development. It includes various activities to facilitate children’s creative thinking, and different types of advice to teachers. Teachers who study the program may become more conscious (TC12).
- Following a certain program, acting within limits is a situation that affects creativity negatively. For this reason, the 2013 preschool program makes limitations to children’s creativity (TC8).

## Conclusions and discussions

The themes derived from the findings of the study and the subthemes related to these themes were discussed by taking into consideration the opinions of teachers and teacher candidates within the light of the related literature. In the first theme, which focused on creativity and the prominence of creative thinking, teachers and teacher candidates mentioned aspects of creativity which emphasized the individual traits of children. The fact that creativity is an important factor in bringing up unique individuals draws attention as an important finding, which was also proved by the research that was conducted by Özkan [8] and which sought answers to the question ‘What is creativity?’. In his study, Özkan [8] reached the conclusion that a majority of teachers defined creativity as the child expressing himself individually, being able to grasp what is authentic, and producing authentic products. Opinions which state that creativity and creative thinking develops the problem-solving skills of children are the items most mentioned by both teachers and teacher candidates regarding the first theme. Opinions which support that children may develop different points of view towards events and situations are supported by thoughts which state that children are able to express their feelings and thoughts authentically. In teachers’ and teacher candidates’ opinions on the prominence of creativity and creative thinking, it is stated that this skill may also positively affect the social development of children. The opinions of the participants which state that training aimed at promoting creativity will lead to positive results both in terms of cognitive development and other areas of development, distinctly overlap with the study conducted by Karadayı [27], in which the researcher states that we should focus on creativity and creative thinking during the preschool stage. In his study, Karadayı [27], studied the effects of creavity education on cognitive processes and the skill to organise emotions, which was given to children aged 5 to 6. It is also stated that creativity education promoted creativity, and the skills to organise cognitive and emotional personality, and reached the conclusion that creativity in the preschool stage was related to both controlled and flexible cognitive skills [28, 29]. Opinions within the first theme which stated that creativity enables children to express themselves individually also draw attention to the social aspect of creativity and creative thinking. In fact, there are other studies which present opinions that children in classes of extremely traditional teachers may experience problems expressing themselves, and that this situation may hinder creativity [10]. If teachers are flexible in their attitudes towards children, and if they pay attention to the individual traits of children, children will be able express themselves easily, and this will strengthen the social function of creativity in children.

Within the second theme, which includes opinions on supporting creativity and creative thinking in children, teachers and teacher candidates mentioned the prominence of techniques that would attract the attention and interest of children during activities conducted with them. Leaving children in the middle of a problematic situation, motivating them to use an object for different purposes or asking them to complete a story are among the practices that may be carried out to support creativity. The participants stated that there are technology-based techniques that can be used to promote creativity in addition to techniques based on communication. Akbaba and Kaya [30], who pointed out that such techniques may be used to enhance the creativity of children by maintaining their interest and curiosity, conducted research with teachers to promote the thinking skills of children. In this research, preschool teachers stated that they mainly used methods and techniques such as hands- on learning, demonstrations, projects, games, and the question and answer to enable students to achieve thinking skills.

There are the opinions of teachers and teacher candidates which state that using different methods (i.e. arts) to promote creativity and creative thinking in the preschool stage will provide positive contributions [31]. In their suggestions regarding in class activities and out of class activities to be conducted with children, teachers have concentrated on conducting activities that offer different options to children rather than standard and monotonous activities. Creativity and creative thinking may yield more development when people break the mold. Teachers developing attitudes that enable their students to express themselves comfortably is one of the most important factors that would eliminate the obstacles hindering creativity [32]. In the findings of the study conducted by Yenilmez and Yolcu [10] regarding the attitudes of teachers in classes on the promotion of creative thinking skills in children, it was stated that children should be given the opportunity to express their thoughts, and that their thoughts should be respected.

It has been emphasized that families should contribute as much as possible during in class and out of class activities. Supporting the child strongly both in the home and at school is a very important factor that accelerates the development of creativity. Chatting to the children about activities during out of class activities, and asking them open-ended questions about the process may enrich their thinking and their mind. Making suggestions to the family to have this point of view while communicating with the child may give the children an opportunity to enhance their creativity throughout the day. The teachers stated opinions which show that if families participate actively during this process, they may provide positive contributions to the child.

Mutlu and Aktan [33] stated that educational programs which are directed towards thinking, and with which the teacher, family and children support and complement each other during preschool education should be prepared. The preschool teachers participating in the study also stated that creativity and creative thinking play an important role in activities which the families do with their children.

It is important to include activities that address different senses for activities conducted in the class, and getting the attention of children. In a study conducted earlier [8] it was found out that a teacher needs to discover the different traits of children by observing them carefully, act as a role model for the child with her / his character, and include music, art, language and game activities in the daily plan that will develop and promote the child’s creativity.

It was stated by teachers and teacher candidates that the preschool educational has a structure that gives the chance to promote creative thinking. The preschool educational program is defined as a child-centred and flexible program which places prominence on research and discovery, and which offers children different activities for learning. The program is a developmental program which places emphasis on creativity as well as family education and family participation [7].

Teachers and teacher candidates expressed that the flexible structure of the preschool educational program enables them to plan according to the individual traits of children and offers the child more freedom. The fact that the program is student- centred may enable the child to display more creative outcomes.

In a research which was conducted to find out the achievements and the indicators in the program in relation to the skills of the 21^st^ century, it was emphasized that 5 achievements within a total of 21 in the cognitive delopment part were found to be in relation with the skills of the 21^st^ century. Similarly, 18 indicators among a total of 113 indicators were found to be in relation with skills of the 21^st^ century. It was stated that 7 of the achievements in the social- emotional development, 5 items in the cognitive development, and 4 of the items in the achievements in linguistic development were parallel to the skills of the 21^st^ century. It is stated that the highest achievements in relation with the skills of the 21^st^ century are the achievements in social- emotional development [34].

It is also aimed to find out the opinions of teachers and teacher candidates for developing creativity and creative thinking in children, tries to evaluate the opinions of participants using a holistic perspective within the context of the preschool educational program. The following suggestions are made based on the findings of the study:

- Problem solving situations that may enable students to display their creativity should be provided.
- Families should participate more in children’s educational process.
- Teachers should include more activities that strengthen the individual traits of children.
- Resources should be provided to teacher candidates to enhance awareness for promoting creativity in the preschool stage.
- Teachers should develop attitudes that are not traditional in the activities conducted with children, and when communicating with the children, as well as taking individual differences into consideration.

As the study was conducted during the Covid-19 pandemic, there was less interaction between the researchers and the participants. Thus, this situation is considered to be the greatest limitation of the study.
